# Hospital and Institutional News

**Published:** 1915-08-14

**Authors:** 


### HOSPITAL AND INSTITUTIONAL NEWS.

### HOSPITAL SECRETARY'S DEATH IN SERBIA.

We regret to record the death on July 31, from typhoid fever, of the Hon. Richard Cecil Frederick Chichester, youngest son of Lord Templemore, who left for Serbia in November last in the capacity of acting honorary secretary to the first hospital unit of the Serbian Relief Fund. Only twenty-five years old, Mr. Chichester, who had been granted the honorary rank of captain in the Serbian Army, soon after his arrival in the country proceeded to Skoplje, where the outbreak of typhus was so severe, and took part in the organisation of his unit, which was certainly of service in controlling the epidemic. In due course, when his part in this work was largely accomplished, he began to tackle the problem afforded by the needs of the civilian population in the south, which was badly in need of an organised scheme of relief. His staff, and the Serbian authorities with whom he came in contact, will miss him, and his death at Nish is a loss not to his family alone, including Lady Paget, his cousin, who was administrator of his unit, but to many others. An Oxford man, Mr. Chichester took his degree in 1910.

### A MENTAL HOSPITAL CONVALESCENT HOME FOR SOLDIERS.

The governors of St. Luke's Mental Hospital, Old Street, London, shortly after the outbreak of war, offered to the Red Cross Society their convalescent home, "Nether Court," near Ramsgate, for use either as a hospital or convalescent home for wounded soldiers. The committee of the Kent Voluntary Aid Detachments having found their hospitals in Ramsgate Town cramped for room, though excellent work, it is understood, has been done during the past ten months, gladly availed themselves of the offer made by the St. Luke's authorities. Since June 25 "Nether Court" has been undergoing alterations for its new purpose; a new block, with operating theatre attached, has been built, and connected up to the house. The whole provides some seventy beds, with ample room for extension should the necessity arise. The change from the crowded town to the beautiful grounds of "Nether Court," of some twelve acres, should prove pleasant as well as very healthy for the soldiers. The hospital is to be formally opened by Lord George Hamilton to-day (Saturday). It is interesting to add that St. Luke's Hospital has a second convalescent branch, "Welders," near Gerrard's Cross, so that the convalescent work of that institution will be little interfered with by the temporary surrender of "Nether Court" to the wounded.

### THE EPPING GUARDIANS AND THE LOCAL GOVERNMENT BOARD.

In our number of July 24 we commented upon the action of the Epping Guardians in dismissing their medical officer after the Local Government Board had ordered his reinstatement. We said the Guardians would find that their decision could not be enforced. It appears that in arriving at it they relied upon the fact that their medical officer when appointed had signed a contract that his appointment should be determinable by a month's notice. The Local Government Board has now written to the Guardians pointing out that "the provisions in the form of contract as to determination by notice only relate to the contract itself, and the contract would have no effect as against the Board's regulations governing the tenure of office of the medical officer, under which his appointment could not be determined except by the Board." This is a most important ruling. Many similar contracts have been entered into by medical officers with their Boards of Guardians, and the fact that the Local Government Board has approved of such appointments on terms which were on the face of them in direct contradiction to the Poor Law Orders has caused surprise. It was generally felt that these contracts could not be enforced, but the verbal quibble by which the Board has now rendered them null and void for practical purposes is not entirely satisfactory. A better and more dignified position would have been to refuse sanction to any contract not in strict verbal accord with the Consolidated Orders. Such action would have prevented any possibility of Guardians and medical officers being placed in a wrong position in their relations the one with the other.

### CHARING CROSS HOSPITAL AND WOMEN STUDENTS.

The project of admitting women students to the practice of Charing Cross Hospital is, we understand, not yet finally decided upon by the Council of the hospital. As far as the Medical School is concerned, this new departure apparently has been decided upon in principle, though, as the hospital is so directly concerned, the scheme cannot take effect until the Council of the hospital have given their full consent. This consent is understood to be complicated by many considerations, and any definite decision cannot be expected at present. Our recent Note which was derived from an unimpeachable source, and was openly discussed by many workers in the hospital, was therefore correct in so far as it referred to the Medical School and not to the Council of the hospital itself. It will be recalled that this Medical School no longer teaches in its own building the subjects of anatomy and physiology, for which purpose it is affiliated to the conveniently situated King's College in the Strand.

### MATERNITY BENEFIT CLAIMS OF SCOTTISH HOSPITALS: GLASGOW CONFERENCE.

Conferences arranged by the National Insurance Commissioners for Scotland have been held recently in Glasgow, Edinburgh, Dundee, and Aberdeen between representatives of approved societies and others interested. The Glasgow Conference, which was held in the Royal Maternity and Women's Hospital, was presided over by Dr. J.

C. McVail, vice-chairman of the Scottish Insurance Commissioners, and among others present were Miss Paterson, Insurance Commissioner, a number of the directors and medical staff of the hospital, and delegates from many of the approved societies. The Chairman referred to the important work carried on by the maternity hospitals, and said there never was a time in the history of this country when the duties which they performed towards mothers and children were more necessary. He urged upon societies the desirability of entering into agreements with the hospitals, and so having the benefit administered in the interests of those members who availed themselves of the services rendered by these hospitals. Dr. Jardine, one of the visiting physicians, addressed the meeting upon the administration of the benefit from the medical point of view. A discussion followed regarding the proposal that approved societies should pay to the hospital a proportion of the maternity benefit received in respect of patients treated in the hospital. At the close a resolution was passed unanimously expressing the opinion of the meeting that it was desirable that approved societies with members in Glasgow and district should enter into a uniform agreement with maternity hospitals in respect of members receiving treatment through the hospitals.

### MILE END INSTITUTION FOR WOUNDED SOLDIERS.

The War Office has made arrangements to take over immediately the workhouse and infirmary of the Mile End Board of Guardians. They are situated in Bancroft Road, and will accommodate over 1,000 wounded soldiers. The patients at present in the institutions will be removed to the Poor-Law institutions at Poplar, Stepney, Whitechapel, and St. George's-in-the-East. Dr. Harley Brooks, the medical superintendent, will remain in charge of the military hospital, with the honorary rank of captain, whilst the workhouse master, Mr. H. G. Chapple, is to be the steward. As in other cases where the military authorities have taken over similar institutions, a number of the inmates who have been carrying out various duties will remain, and be maintained at the cost of the military authorities, who will allow each of them 2s. 6d. a week pocket-money.

### CANADIAN CONVALESCENT HOMES.

We lately reported the opening of the New Zealand hospital for convalescent soldiers, one of the aims of which is to provide a special institution for the benefit of New Zealand wounded, so that they may have as much a home atmosphere as is possible for them in the Mother Country. It is now satisfactory to learn from the Toronto correspondent of the *Times* that the Government is arranging for convalescent homes to be established in the Dominion, to which Canadians at present accommodated in British hospitals will be moved, as soon as they are fit for the journey and the new institutions are ready. It is understood that the wounded so transferred will be placed on full pay in hospitals under military regulations until they are wholly recovered or are at length formally discharged on pension. From the point of view of the Canadian wounded the new development is likely to be much appreciated. Canada is the nearest of the Colonial Dependencies, and this fact, presumably, may mean that a similar plan is found impracticable in Australia and New Zealand.

### THE HUNT FOR THE EARLY CASE.

The work of Insurance Committees tends to become more and more varied as the machinery begins to run more smoothly and time is found to deal with the human problems that continually crop up. That the Act as a whole is still causing dissatisfaction in more than one direction and that in some areas there is a certain amount of muddle cannot be denied, but it is a fact that many Insurance Committees are performing their tasks as efficiently as is possible, and aiding in various ways insured persons to obtain the greatest benefits by the most profitable and timely methods. The sanatorium benefit, as might be expected, entails a great amount of work, and the sub-committees dealing with tuberculous persons are always kept busy. A recent meeting of the Oldham Insurance Committee might serve, presumably, as a typical example of such meetings. Among the matters brought forward was a complaint of delay in the diagnosis and reporting of a case of phthisis. This question was not finally threshed out, but the complainant stuck to his guns and urged that greater watchfulness and full inquiries when necessary would stimulate the doctors to make earlier diagnosis and thus give patients a better chance. Whatever the truth about the allegations regarding the case mentioned, the circumstances are such as would be very unlikely to recur, and at the present time many people think that the hunt for the early case of pulmonary tuberculosis is being conducted in most areas with sufficient zeal or even, it is whispered, with excessive energy.

### THE SANATORIUM "DEATH SENTENCE."

Another matter which was brought up at this same meeting last month by the tuberculosis officer, Dr. J. B. Wilkinson, is one of some interest as similar circumstances obtain in other districts. Where there are two separate sanatoria to which patients are sent, it usually happens that for some reason a majority of the advanced cases is to be found in one institution, which then rapidly acquires a bad name. So much is this sometimes the case that patients regard an invitation to that institution as a death sentence and refuse to avail themselves of the "benefit." But the facts are scarcely ever thus, and even if a good many advanced cases do get to one particular sanatorium there are often also observation wards for doubtful or special cases in the same institution. It becomes, therefore, of great importance, as Dr. Wilkinson pointed out, to prevent any impression gaining currency which should suggest that only fatal cases were sent to a certain institution, unless, of course, this was intended from the first as a segregation hospital or home for the dying pure and simple.

### THE FUTURE OF DARTFORD TRAINING COLLEGE.

All hospital officers, and many nurses, who have appreciated the services rendered to science, therapeutics, and training by the Swedish Physical Training College, founded by the late Madame Osterberg, will learn with interest of the arrangements made by her, just before her death, for the continuation of the work of the college. On this subject we learn from the Board of Education that when, about eighteen months ago, Madame Osterberg desired to relinquish the active direction of the Swedish Physical Training College at Dartford, of which she was the principal and founder, she wished, in the national interest, to secure the continuation of the work which had been so successfully established and developed. She offered, with the full approval and sympathy of her husband, Dr. Edwin Osterberg, of Stockholm, to transfer her college to the Government. For reasons in no way connected with the college, remarks the Board of Education, it was found impracticable to accept the offer, and Madame Osterberg was advised to create a trust. Almost her last act before her death was to sign the trust deed, vesting her property in a trust with the object of carrying on the college in the national interest on its existing lines. The trustees are Dr. Addison, M.P., Mr. Waldorf Astor, M.P., Sir George Newman, M.D., Lady Salisbury, and Lord Shaw of Dunfermline. While the ultimate control of the institution rests with the trustees, the general management and working will be placed in the hands of an executive committee of ten persons representative of various official and other bodies concerned with the Physical Education of Women.

### THE LATE MADAME OSTERBERG'S WORK.

In view of her death at the age of sixty-five, it is appropriate, therefore, to recall the beginning of the movement, of which Madame Osterberg was the head, for Swedish physical training. A Swede by birth, who settled in London some forty years ago, she was appointed superintendent under the old School Board, a post she occupied for five years, until in 1885 she opened at Broadhurst Gardens, Hampstead, the first college in this country for training teachers in Ling's Swedish system. This school, we need not remind our readers, became the recruiting centre to which heads of schools applied from all over Europe for trained teachers. Indeed, in ten years' time the work so much developed that a move had to be made to larger premises at Dartford Heath, and twenty years—that is to say, till the time of her recent death—were spent in carrying on the work. The period was one of growth and continued expansion, a research laboratory, for instance, being added of late years. As readers of the previous paragraph will appreciate, her offer of the college to the Board of Education was a generous one, since it is understood that a sum of £30,000 had been tendered to her for it. Her last two years were spent in the country of her birth, where, in addition to carrying on what may be termed her hygienic propaganda, she gave a course of lectures to help to prepare her sex for the enfranchisement which she expected them to receive at an early date. These lectures, which covered the whole ground of politics and sociology, in Aristotle's sense of the former word, attracted men as well as women listeners. Her last foundation was, we believe, that of the Agricultural College for Women at Bästad, where an institution somewhat on the lines of Swanley for the training of women in gardening and horticultural pursuits was established by her in 1914. It will be noted that she belongs to the rare type of woman organiser of which the nineteenth century provided the supreme example, for the modern world, in Florence Nightingale.

### THE SUPPLY OF SALVARSAN.

The effects of the injection of English-made salvarsan were disclosed at an inquest on Saturday at St. Pancras on Lieutenant James V. McIntyre, of the London Scottish Rifles. On four different occasions during the past four years injections of salvarsan had been given him, under medical advice, the last time being four days before his death. He had arranged to take a private room at the Italian Hospital, Queen Square, Holborn, so that Dr. Atal, the house surgeon, might make another injection. On Tuesday evening he went to the hospital, when Dr. Atal injected the drug, whereupon the patient suddenly died. Dr. Atal informed the Coroner that he had administered the drug in over 1,000 cases. Previous to the war it was obtained from Germany, but now it was made in England and France. In 800 cases he had administered German-made salvarsan, in 100 English, and in another 100 French salvarsan, and had not had any ill-effects. He found, however, that in the use of English salvarsan the patient was more likely to have vomiting. Dr. Bernard Spilsbury, pathologist at St. Mary's Hospital, said that death was due to asphyxia, whilst deceased was suffering from disease of the brain, and, in his opinion, the injection of the drug had no part in causing death. Owing to his condition he was liable to expire suddenly if he received any shock, and the witness believed that the fatal seizure arose from the deceased being in a state of nervous alarm at the operation. He would certainly not have been able to withstand the shock of war.

### THE EMPIRE HOSPITAL AND THE WAR OFFICE.

A joint committee has now been formed to manage the Empire Hospital, which, as noted in our issue of July 24, has been taken over by the War Office as an extension of Lord Knutsford's scheme for the provision of specialised hospital treatment for cases of organic nerve injury. The committee consists of three members of the management board of the Palace Green Institution and the directors of the Empire Hospital. The control of the hon. medical and surgical staff, which includes some of the leading neurologists in the country, is vested in Lord Knutsford's committee, acting under the Director-General of the London District; while the appointment and dismissal of the nursing and domestic staff, as well as the control of the finances, remain with the directors of the Empire Hospital. The Empire Hospital is intended to accommodate cases of organic nerve lesion, but, pending the time when it will be possible to utilise all the beds for this purpose, accommodation will be devoted to the class of cases hitherto sent, and these will remain under the supervision of the Army Medical Service. A third hospital is being established by Lord Knutsford for the treatment of the more acute mental cases. Past experience seems to suggest that the ample accommodation provided for these types of nerve injury is not, perhaps, likely to be fully required.

### THE MATERNITY WARDS AT WANDSWORTH.

THE Wandsworth Board of Guardians are to be congratulated on receiving unstinted praise from Miss Stansfield, inspector of the Local Government Board. Recently she visited the maternity wards of the institution, and in a report to the Board stated that she was very much impressed with the great improvement that had taken place in these wards. On the occasion of her previous visit they had been far from satisfactory, but now she was able to state that they were second to none in the Metropolitan district. Indeed, she did not remember such a rapid improvement taking place in any other Union in London. Naturally the Wandsworth Guardians felt encouraged at this high praise, and, in case there should be any complaints in the near future, decided that Miss Stansfield's letter should be recorded on the minutes. The improvements, however, must be largely credited to Dr. Dodson and the matron. The staff, having leisure to turn their attention to the maternity wards after getting other sections of the work into proper order, have initiated reforms in the maternity wards, beneficial alike to the staff and the patients, which make them at present a model and standard for what a Poor-Law infirmary maternity block may be.

### MILITARY RANK FOR A LADY SUPERINTENDENT.

FRANCE, the country of ideas, has accorded the rank of Médecin-Major to Dr. Helen Sexton, Directress-in-Chief of the Australian Hospital at Auteuil. According to the first account, which came through Reuter, the staff, consisting of women, has been organised by two Melbourne ladies, Mrs. Smith and Mrs. Blackwood, who have financed the institution.

### A BRITISH STAFF UNDER THE RUSSIAN RED CROSS.

THE magnificent steadfastness with which the Russian retirement is apparently achieving its aim—namely, the strategic defeat of the German Army through the maintenance of the front of its own forces in the face of their attempts to pierce and separate our Ally—is enough to secure sympathetic attention for the appeal (signed by Lord Cheylesmore, chairman of the Brompton Hospital, and others) on behalf of the proposal to establish a hospital, with a British medical and nursing staff, under the Russian Red Cross. To carry out the promoters' intention—namely, to equip and maintain for one year a hospital of 200 beds—the sum of £30,000 is asked for. The Anglo-Russian Committee in London is associated with the Special Committee, of which Lord Cheylesmore is chairman, entrusted with the task of carrying the proposal into effect. Information is obtainable on all points from the honorary secretary, at the address of the Anglo-Russian Hospital, 116 Victoria Street, Westminster, S.W. Monthly subscriptions over a period in place of a donation are hoped for to make easier the task of maintenance.

### A MOTOR CABMAN VERSUS A MEDICAL PRACTITIONER.

A CURIOUS case involving a doctor, in the capacity of what used to be called a "fare," was heard at Marylebone Police Court last week, when Dr. E. J. Haynes, of Crediton, near Exeter, was summoned by a taxi-cab driver for the recovery of £5, the fare, as was alleged, for a drive begun at 8 A.M. in Elgin Avenue, Maida Vale, then "all over the City," ending up, for the time being, at Knightsbridge. On the day after this drive had been taken Dr. Haynes, the taxi-cab driver contended, was called for at the same address, and in the course of the drive the fare, which had amounted to £2 16s. at the end of the first day, had risen to £5. The doctor, saying that he had no money, promised to forward it, and in the meantime presented the driver with an I O U. The magistrate, Mr. Paul Taylor, in making an order for the payment of the £5, with 17s. costs, informed the cabman that, if the money was not paid, he could put in a distress.

### MR. PUNCH AS HOSPITAL CRITIC.

WE are glad to see that Mr. Punch keeps his vigilant eye not merely on the verbal slips or examples of unconscious humour, which in a newspaper-ridden industrial population must always be numerous, but also that (at his best) he turns his critical spirit of comedy, after the fashion of Molière, on more important things. Thus, in Wednesday's issue, Mr. Punch quotes the following passage from the *Sydney Evening News*: "The *Gunaandal* came in on Saturday afternoon with twenty-five baskets of fish, averaging about 65 lb. each, and only about 5 per cent. were not edible. These" (*sic*) "were distributed among the hospitals." Mr. Punch comments on this in these judicious words: "On the theory" (he presumes) "that as the patients are ill, they may as well be very ill." Thus wisdom is justified of her children, and *Punch* has always been regarded as the wisest, that is etymologically, remember, the wittiest of the gods. What are the people of Sydney doing to allow "not edible" fish to be distributed at all to their inhabitants, much more to the patients in the hospitals of their city? or, if the fact of the destination of the inedible fish is misreported, to tolerate such shocking grammar? Since bad grammar is the product of loose thinking, in either event Mr. Punch has shown that the good folk of Sydney are in a parlous state. Perhaps, however, the sub-editor of the *Sydney Evening News* has joined the Forces; but, if he is one of our heroes, who have they allowed to take his place?

### PHTHISIS AND THE SANITARY INSPECTOR.

From time to time we have recorded the various views expressed as to the adequacy of the domiciliary treatment of cases of tuberculosis, and of the best means of improving the system, which is inevitably open to many defects from which institutional treatment is free. Only a fortnight ago we remarked that any community which has provided insufficient beds for the accommodation of the advanced case was merely trifling with the tuberculosis problem. At the other end of the scale is the method of domiciliary treatment, for the early case, and in this matter medical officers of health and tuberculosis officers are at once brought up against home conditions, which, translated into practical politics, mean the housing question. What this involves from the point of view of the medical officer of health is clearly brought out in Dr. Hyslop Thomson's report on the public health of Hertfordshire. As deputy county medical officer of health the problem has presented itself to him, with the result that he reviews the question as follows: "To secure a result commensurate with the cost of the organised efforts . . . improvement in the home conditions of the people must proceed *pari passu* with treatment of existing cases of the disease." To remedy existing defective conditions and to maintain an adequate standard "frequent and methodical inspection is necessary." The recommendation for the appointment of a county sanitary inspector to co-operate with the district inspectors and to keep the council in constant touch with the housing conditions of the working-classes is, therefore, re-emphasised and repeated. In short, the two ultimately economical methods of controlling tuberculosis are the provision of beds for all advanced cases, and the adequate inspection and maintenance of a high standard of housing. Every county council which does not throw its weight into the scales on the side of these two reforms is unworthy of the confidence of ratepayers whose interest it is supposed to serve by maintaining a high standard of public health throughout its area.

### THE HOSPITAL BUTTON.

We all know the attachment which is felt by the wounded for their war trophies, particularly for those which have a foreign air, such, for instance, as a German helmet. Again a wounded man, who is grateful to the sister or nurse of the ward in which he is placed, will often show his regard by the present of a pair of buttons, which are turned, in due course, into a pair of cuff-links, or some useful, and ornamental, article of female attire. This apparently small matter has assumed significant administrative proportions at least in one institution, as the following decision will show. The Scottish Women's Hospital at Royaumont is now apparently in need, for its complete equipment, of several gross of Union Jack buttons. The reason appears to be that the hospital is popular among the French wounded, who, therefore, on leaving, seek a souvenir of the institution. Thus the organisers of the Scottish hospitals propose that a special button should be designed bearing the name "Royaumont," and the Union Jack. It is suggested that the button should have the form of a medal hung from a piece of tartan, and be inscribed "To our Allies from their friends at Royaumont." Thus the hospital badge is quite outclassed by the ingenious hospital button!

### IRISH GUARDIANS, EMPLOYEES, AND WAR WORK.

In considering the allocation of sums received for the maintenance of soldiers, and payments to officers in connection with the military hospital, the finance committee of the Belfast Board of Guardians have made certain recommendations in connection with the matter of extra payments to various members of the staff and institutional employees. The sum of £233 has been spent on alterations to the hospital, and, after interviewing the visiting medical officers, Dr. Hall and Dr. McLiesh, the committee recommend that a payment of 4s. per head per week should be made to them for the patients each had treated. The residents are also recommended to receive "any payment that may be necessary." It is interesting to note that, while the Guardians received payment at the rate of 17s. 6d. weekly per head till March 31, and 21s. per head from April 1, 1915, the balance they anticipate, after the above payments have been made to the staff, will, they calculate, recoup them for the cost incurred in the maintenance of soldiers and expenditure on alterations. Various sums are also to be paid to the lady superintendent and the nursing and domestic staffs for the period during which such patients may be treated in the military hospital.

### AN UNWRITTEN CHAPTER IN HOSPITAL HISTORY.

In view of the article which we published last week on the interesting chapter of hospital history formed by the development and sudden expansion of the Voluntary Aid Detachments, as set forth in the Kent and Devon County Voluntary Aid Detachment histories, the comparative cost of running the Wiltshire Voluntary Aid Detachment institutions is worth recording. According to a statement issued by the committee of the Trowbridge Red Cross Hospital, it appears that claims for grants have been made on a sliding scale between the extreme of 2s., rising to the largest claim of 4s. per head. The Trowbridge institution, which claims the distinction of asking for the lowest grant of any institution in the county, attributes its favourable position on the list apparently to the numerous gifts in kind which it continues to receive in response to the committee's weekly appeal. With the technics of the Valuation of Gifts in Kind, from the point of view of the hospital accountant and statistician, we deal in a remarkable article elsewhere in this issue; but, though many points would have to be inquired into before the precise significance of these figures would be apparent to the administrator, the publication of the table appears to show a friendly rivalry, and points to the need for working out the statistical cost of maintenance and administration in the Voluntary Aid Detachment institutions of each county; for their work has been significant enough to demand its own volume in the library of hospital history on the war, which will eventually, we hope, find its Gibbon, or its Froude.

### THE INSTITUTIONAL SIDE OF PHILANTHROPY.

Manchester and Salford possess a Boys' and Girls' Refuges and Children's Aid Society, which has grown to a considerable size in the forty-six years of its existence. Like most of those philanthropic bodies which attempt to tackle their work seriously, the society has now become impressed with the need for providing institutional treatment for its anæmic or otherwise debilitated children. Last Saturday its purpose was achieved to a large extent by the opening of a house converted now into the society's second convalescent home, near Old Colwyn Bay, in the presence of the Lord Mayor of Manchester, who performed the opening ceremony. Mr. T. R. Ackroyd, the hon. secretary, pointed out in an informative speech that the cost of maintaining the 4,381 children who were under the society's care last year was £12,000, all of which, he said, was voluntarily subscribed. To this he added that the children contributed £3,200 out of their own earnings. Those who will have the benefit of the new Tanllwyfan Convalescent Home will be drawn, he said, from the poorest part of Manchester and Salford. The society's first convalescent home is situated at Lytham, and we congratulate them on this addition to their equipment, for experience shows that there is no more profitable discipline for lay or religious philanthropy than that provided by institutional responsibilities.

### AUGUST 12.

The sportsman's annual calendar, so far as the shooting season is concerned, begins as we go to press this week. The grouse shooting season, therefore, coincides with the appearance of this issue, and hospital managers will note with interest the practical attempt made by Commander Graham, R.N.V.R., to secure a distribution of game to the different military and naval hospitals in such a way as to avoid a glut at one hospital and an absence of any gifts at another. He has, therefore, announced that the executive committee of the Scottish branch of the Red Cross Society has agreed, on his suggestion, to form a special department, which he describes as a game clearing-house, where a receiving and distributing depôt for game has been established. The address is 48 West Nile Street, Glasgow. Commander Graham also appeals to proprietors of moors to support his scheme, and we see no reason why it should not be adopted in the different counties or commands in England also. Last year stories reached us of a glut of pheasants in certain West Country V.A.D. hospitals, where the staff lived on this bird for so long, in their endeavours to make use of those birds which remained over after the patients' needs had been supplied, that the taste of pheasant became almost nauseating to them. Even then many birds had to be thrown away. As August 12 is very quickly followed by September 1 (partridges) and October 1 (pheasants) we suggest that there is still time to organise a similar scheme south of the Tweed. If the chance be lost, much needless waste is again extremely probable.

### A RUN OF LUCK TO LONDON HOSPITALS.

The voluntary hospitals, or at least certain of them, among which is noted again the Royal Free, Gray's Inn Road, have been receiving a remarkable run of legacies. Mr. J. C. Eno's bequests we recorded last week, and again in this issue the bulk of a fortune of £165,000 goes to two London hospitals. Mr. George Patrickson, whose estate has been valued at this sum, after bequeathing £10,000 to the Cremation Society and £4,000 to the Ulverston Cottage Hospital, left the residue of his property to St. George's Hospital and the Royal Free Hospital in equal shares. The residue is expected to amount, after the payment of duty, to over £100,000. It is noteworthy that his choice fell on the two London hospitals which are perhaps just now, if we except the Westminster, most deeply interested in extension or migration schemes. St. George's wishes to go, and apparantly cannot; while the Royal Free is deeply concerned in the immense development of the London School of Medicine for Women, attached to it, which has received such an immense impetus because of the war.

### THIS WEEK'S DRUG MARKET.

Business has now completely recovered from the holiday and is just as active as ever. In normal times the drug market is usually very quiet at this season of the year, but such is not the case at the present time, for there is no cessation in the demand for drugs for the treament of wounded soldiers. The most noteworthy price change that has occurred since those noted in last report is an advance in the price of iodine, and consequently of potassium iodide, sodium iodide, and iodoform; the advance came as a surprise. The abnormally high quotations for cod-liver oil are still maintained; it is difficult to know what advice to give to intending purchasers, but it might be as well to follow the example of experienced buyers who are holding off the market for the present. Quinine is slightly dearer. The prices of practically all the synthetic drugs have an upward tendency, and acetanilide, which was slightly lower in price a week or so ago, is now inclined to be dearer again. Opium is steady, and morphine and codeine are unchanged and in large demand. Senega is slightly dearer. Ergot is rather lower in price.

